# Sex-specific sterility caused by extreme temperatures is likely to create cryptic changes to the operational sex ratio in *Drosophila virilis*

**DOI:** 10.1093/cz/zoaa067

**Published:** 2020-12-14

**Authors:** Benjamin S Walsh, Natasha L M Mannion, Tom A R Price, Steven R Parratt

**Affiliations:** Institute of Integrative Biology, University of Liverpool, Crown Street, Liverpool, L69 7ZB, UK

**Keywords:** climate change, reproduction, sexual selection, fertility, climate change, reproduction, sexual selection, fertility

Climate change is increasing the frequency and severity of short-term heat shocks that threaten the persistence of natural populations. However, most work addressing the evolutionary consequences of anthropogenic environmental change has focused on natural selection, with less attention paid to the impacts on sexual selection. The conditions under which sexual selection operates is a topic of debate, but a generally observed pattern is that the operational sex ratio (OSR) of a population is key to determining both the extent of competition for fertilizations and the scope for mate choice (Weir et al. 2011). Therefore, if high temperatures affect the ratio of reproductive males to females in a population, this could influence sexual selection. Sub-lethal temperatures can sterilize individuals from a range of biological systems, including plants, insects, corals, birds, and mammals (reviewed in Walsh et al. 2019a). If high temperatures affect reproduction in one sex more than the other, this may create cryptic shifts in the OSR of a population (Petry et al. 2016). However, although fertility loss at high temperatures is generally thought to be more common in males than in females (Iossa 2019), very few studies measure fertility in both sexes under identical conditions (Walsh et al. 2019b). Where sensitivity to temperature has been observed to vary between the sexes (Janowitz and Fischer 2011; Zwoinska et al. 2020), the effect on population sex ratios has not been considered. Furthermore, natural selection, sexual selection, and population dynamics are more likely to be affected by biased sex ratios if sterility is long-lasting. However, to date, patterns of sexually dimorphic heat-induced sterility have not been shown over organisms’ reproductive life spans. Here, we aim to test whether heat stress differentially affects male and female fertility in the cosmopolitan fruit fly *Drosophila virilis* and if this creates cryptic bias in population sex ratios over time. Specifically, we hypothesize that pupal heat stress will significantly delay adult sexual maturation and that this will be more severe in males compared to females under identical conditions. To do this, we exposed pupal *D. virilis* to a sub-lethal heat shock of 38°C for 4 h to simulate the peak of a mid-day heat wave. We chose to heat pupae because they are immobile and cannot behaviorally escape heat stress in nature. We subsequently examined both complete sterility and pupal offspring production over an ecologically realistic lifespan in both males and females. We combine male and female time-series data to predict the effect of heat-induced sterility on the OSR, and discuss its potential consequences on sexual selection. Detailed methods are described in the Supplementary Materials.

We found that the rate at which newly eclosed *D. virilis* become fertile is significantly influenced by the interaction between sex and temperature. While female fertility is not significantly affected by heat stress, male sexual maturation is significantly extended if they are exposed to 38°C as pupae (Cox proportional hazard test interaction term: hazard ratio (HR) = −1.4866, $\chi^{2}{}_{1}=16.275$, *P *<* *0.001; Figure 1A and B). Furthermore, we found that the proportion of individuals that never produced offspring was predicted by a significant interaction between sex and treatment, wherein males exposed to heat stress were more likely than controls or females in any heat treatment to be rendered permanently sterile (χ^2^_1_ = 5.657, *P *=* *0.017; Supplementary Figure S1). This is a relatively small effect, showing that most males recovered fertility at some point during the experiment. We found that control males reached sexual maturity 7 days post eclosion, in line with previous observations. This results in an observed OSR for control males and females to stabilize at 0.5 from that point 7 days onwards (Figure 1c). In stressed males and females, however, the sterile males prevent the OSR from reaching 0.5 over the 17-day duration of our experiment. This results in an observed female bias in the sex ratio when flies are heated as pupae (Figure 1C). In males, pupal heat stress significantly reduced pupal offspring number by 58% (estimate= −0.870, *t_59,1_* = −3.925, *P *<* *0.001; Supplementary Figure S2A), and variation in the number of progeny from heated males was significantly lower than that in benign males (*F*-test: *F*_59, 1_ = 2.837, *P *<* *0.05). In females we find no significant effect of temperature stress on pupal offspring number (estimate = −0.081, t_69,1_ = −0.928, *P *>* *0.05, Supplementary Figure S2B), and there was no significant difference in variation of offspring number in the 2 female treatments (*F*-test: *F*_69,1_ = 1.105, *P* > 0.05).

**Figure 1.**
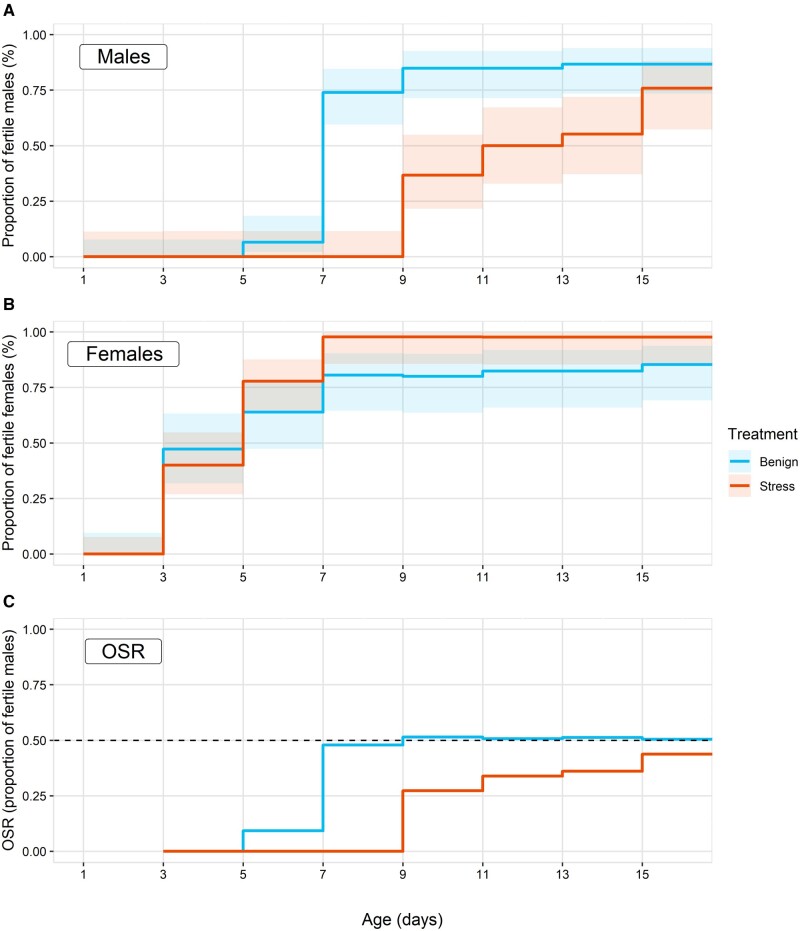
Cumulative proportion of (**A**) male and (**B**) female *D. virilis* that were qualitatively fertile at each time point post-eclosion. Individuals were either kept at benign temperatures (23°C) or stressed (4 h at 38°C) during the pupal stage (Sample sizes (number of individuals): benign males = 45, stressed males = 29, benign females = 35, stressed females = 45). Both sexes eclose as sexually immature adults and become fertile as they sexually mature. This rate of maturation is significantly slower in males that have been exposed to 38°C heat shock as pupae. Error ribbons represent 95% confidence intervals estimated from survival model fits. (**C**) Estimated OSR based on fertility patterns in A) and B) (OSR, proportion of fertile males as the proportion of all fertile adults). Horizontal dashed line represents a 1:1 sex ratio.

A small but significant proportion of males was permanently sterilized by pupal-heat shock (∼25%). A much larger proportion of males was rendered temporarily sterile because heat stress slowed post-eclosion sexual maturation, doubling maturation time for some males. This delayed sexual maturation due to heat stress supports findings from other *Drosophila* species (Jørgensen et al. 2006). In contrast, females showed no significant loss in fertility nor offspring production when stressed at sub-lethal temperatures. Heat-delayed reproductive maturation in males but not females induces a significant period of male sterility during which the population OSR is skewed. A major question is whether our results capture what we would expect to see in natural populations that experience extreme temperatures. Under benign temperature conditions, male *D. virilis* eclose as sexually immature adults and become fully fertile over 5–7 days. We tracked fertility for up to 17 days, and half of heat-stressed males did not become fertile until 11 days post eclosion. Best estimates suggest *Drosophila* rarely survive beyond a few weeks as adults in nature (Powell 1997), so a loss of fertility for even a few days could seriously impact individual fitness. This effect would be particularly acute in populations and species whose life history and phenology permit limited time windows for reproduction. Further, in our study, focal flies are given optimal conditions and opportunity to reproduce (multiple mates, no competition, *ad libitum* food, and a stable benign environment as adults). Despite these ideal conditions we still see significantly higher permanent sterility in males that experience heat stress compared to control males and both female treatments. These results demonstrate that sexual dimorphism in sub-lethal thermal tolerance traits has the potential to shift the OSR of heat-stressed populations across time. This would result in heavily female-biased populations in which the availability of fertile mates is scarce over shorter periods in nature, possibly driving plastic or evolutionary changes in reproductive behavior. Whether the OSR shifts we see in our data would be sufficient to drive evolutionary rather than plastic responses, and whether responses would be through sexual or natural selection are open questions. Ultimately the selective strength of OSR biases will depend on both the short-term duration of sterilizing events and the long-term frequency of such events.

A key finding in our data is that shifts in the OSR happen at sub-lethal temperatures, and so are not reflected in the observable adult sex ratio. This is in contrast to observable temperature-driven sex ratio shifts in species with temperature-dependant sex determination. Therefore, cryptic sterility presents a problem for biologists trying to link observable sex ratios in nature with evolutionary processes. Further, if cryptically sterile males behave like fertile males this could influence female mating behavior. For example, heat-sterilized *Drosophila pseudoobscura* males continue to court and mate females normally, which forces females to remate to become fertilized (Sutter et al. 2019). Increased mating rates can in turn result in female harm through direct damage, ejaculate proteins, or sexually transmitted infections, all of which have been implicated in driving sexual and natural selection. Measuring how heat-induced cryptic sterility biases sex ratios and how this influences sexual selection, natural selection, and population dynamics, will inform our understanding of how climate change affects natural populations.

## Funding

B.S.W. was funded by the “Adapting to the Challenges of a Changing Environment” (ACCE) Doctoral Training Partnership, which is itself funded by the Natural Environment Research Council (NERC) [NERC grant NE/P002692/1 to T.A.R.P.].

## Supplementary material

Supplementary material can be found at https://academic.oup.com/cz.

## Authors’ contributions

B.S.W., T.A.R.P., and S.R.P. contributed to conception of the work. The methodology and data collection were contributed by B.S.W., N.L.M.M., and S.R.P. Data curation was done by B.S.W. B.S.W. and S.R.P. contributed to data analysis. Original drafting of the article was done by B.S.W., T.A.R.P., and S.R.P. All authors reviewed and contributed to editing of the manuscript .

## Data and materials availability

All data and analysis R code has been deposited on Dryad and can be found at: at https://doi.org/10.5061/dryad.dv41ns1w8

## Supplementary Material

zoaa067_Supplementary_Data
