# Supplementary material for: Sex-specific sterility caused by extreme temperatures is likely to create cryptic changes to the operational sex ratio in Drosophila virilis
Source: Curr Zool. 2020 Dec 14;67(3):341–3. doi: 10.1093/cz/zoaa067 (PMC8489007; doi:10.1093/cz/zoaa067)
Supplement: zoaa067_Supplementary_Data [file zoaa067_supplementary_data.zip › supplementary materials.pdf]

Sex-specific sterility caused by extreme temperatures is likely to create cryptic changes to the operational sex ratio in *Drosophila virilis*

Benjamin S. Walsh*, Natasha L. M. Mannion, Tom A. R. Price & Steven R. Parratt

Institute of Integrative Biology, Crown Street, University of Liverpool, Liverpool, L69 7ZB, UK

*Address correspondence to Benjamin S. Walsh. E-mail: bwalsh@liverpool.ac.uk

Handling editor: Natalie Pilakouta

Received on 30 July 2020; accepted on 20 October 2020

Keywords: climate change, reproduction, sexual selection, fertility

Materials and Methods

Animal stock maintenance

Stocks of *Drosophila virilis* (Cambridge Fly Facility StrainvS-4, isolated in 1991), were kept in a temperature-controlled room at 23°C, 12:12 L:D and ambient humidity, selected based on observations of when the laboratory populations are most stable. Stocks were maintained at moderate density (50 – 100 flies per 300ml bottle culture) on 'Propionic' medium (10g agar, 20g yeast extract, 70g cornmeal, 10g soya flour, 80g malt extract, 22g molasses, 14ml 10% nipagin, 6ml propionic acid, 1000ml H_2O). Ovipositing adults were tipped to new food every week to keep density relatively constant and prevent overlapping generations. Ovipositing adults were replaced with younger adult flies every 4-6 weeks.

Assaying for sexual dimorphism in thermally induced sterility

D. virilis are not sexually mature when they first eclose, males and females reach maturity 6 and 9 days post-eclosion respectively (Pitnick et al. 1995). We first hypothesise that pupal heat-stress will induce a significantly longer period of complete sterility post-eclosion compared to controls. We further hypothesise that this effect will be sexually dimorphic, in that males will be rendered completely sterile for longer than females under identical pupal heat-stress conditions.

We based our assay for temperature induced sterility on Jørgensen et al. (2006), wherein the authors demonstrated that a 4-hour heat-stress of adult *D. buzzatii* suppressed male fertility. Whilst other work has used life-long stress to sterilize *Drosophila spp.* (Rohmer et al. 2004), a 4-hour shock arguably better captures ecological reality by replicating the peak of a heatwave in the middle of the day. Unlike Jørgensen et al. (2006), we applied heat stress to

early-stage pupae, and tested both males and females. We use pupae to test if early-life heat stress can completely prevent reproduction in adults. Pupae are sedentary and so would be unable to behaviourally thermoregulate in nature, unlike adults.

Focal animals for our experiments were collected directly from stock bottles within 24 hours of pupation and allocated at random into groups of 30 in fresh 25 x 95mm plastic vials containing 25ml standard 'ASG' medium (10g agar, 85g sucrose, 20g yeast extract, 60g maize, 1000ml H_2O, 25ml, 10% Nipagin) to prevent desiccation. We did not directly control rearing density in our flies, but pupae were taken from stocks of similar age and were randomly allocated across treatments to homogenise any variation due to density during rearing. We use ASG because pilot experiments showed that the propionic acid in the 'Propionic' food reduces survival of pupae when heated. Immediately after collection, 180 pupae (3 vials containing 30 pupae per treatment) were randomly assigned to pre-heated water-baths at either a benign (23°C) or a stressful temperature (38°C) for 4 hours between 10am to 2pm. Preliminary experiments showed 38 °C to be the highest temperature at which we do not see significant heat-induced mortality (Supplementary Figure S3, Sample sizes: 23°C= 81, 37°C= 60, 38°C= 60, 39°C= 80, 40°C= 80, 41°C= 80.).

Following heat-stress, vials were returned to temperature-controlled rooms set at benign temperature (23°C) and flies were observed daily for eclosion. In total, 35 female and 45 male adults eclosed from 'benign' pupae, and 45 female and 29 male adults eclosed from pupae stressed at 38°C. "The sex ratio of emerging individuals did not significantly deviate from the expected 1:1 at either 23°C (exact binomial test: $p = 0.38$), nor 38°C (exact binomial test; $p = 0.14$). At eclosion, all flies of both sexes were isolated as virgins into individual vials containing 'Propionic' food and four sexually mature virgin partners from the opposite sex.

We used four partners as it reduces the risk of false negative fertility scores due to failures to copulate through mate-choice, or any inherent sterility in the non-focal flies. Mating partners were reared from stock populations at 23°C and were 7-9 days post-eclosion to ensure sexual maturity (Pitnick et al. 1995). All five flies in each vial (one focal male with four females, or one focal female with four males) were tipped into a fresh vial of 'Propionic' food every 2 days for 15 days and all flies were discarded on day 17 (resulting in 8 vials of offspring per focal fly). Vials for all focal individuals from every time point were kept at benign temperatures (23°C) for days 1 to 10 of the experiment, and were then transported to fluctuating room temperatures (approximately 18 – 22°C, UK room temperatures in early March 2020) for days 11 onwards because of a shift to home-working due to the 2020 COVID-19 pandemic. This change in rearing temperature was applied to all treatments equally, and given that *D. virilis* is a hardy cosmopolitan species associated with human habitats (Mirol et al. 2008), it is unlikely this had a significant impact on individuals' ability to copulate, oviposit nor on offspring development.

We scored fertility (either completely sterile or able to sire at least one offspring) by observing the presence/absence of larvae directly in vials or by identifying the distinctive larval tracks in the food. We counted offspring production as the number of pupal cases adhered to the vial wall on the first days that adult F1 emergence was observed for that time-point. Pupal case number rather than true adult progeny counts were used due to practical limitations of home-working, but *D. virilis* lay offspring in relatively low density and almost always pupate away from their food which facilitates accurate counting.

Statistical analyses

All statistical analyses were completed in R (version 3.5.0), using the packages: binom (Dorai-Raj 2014), car (Fox 2011), and “ggplot2” (Wickham 2016) “survival” (Therneau 2015),.

a) Fertility over time

We analysed the effect of heat stress on fertility over time with inverse Cox proportional hazard survival analyses (using the “survival” package (Therneau 2015)). This allowed us to model the time in days post-eclosion until focal individuals become fertile. We fit the time point at which fertility (scored as the presence of offspring) was observed as our response variable with sex (male or female), heat treatment (benign or stress), and their interaction as independent variables.

Some individuals never produced offspring during the experiment and so were scored as ‘permanently sterile’. To determine if heat-stress increases permanent sterility, we performed a logistic regression with permanent sterility as a Bernoulli response variable and sex (male or female), heat treatment (benign or stress), and their interaction as explanatory variables. Significance of predictors and interactions was determined with Wald Chi^2 tests implemented in the `car` R package (Fox 2011).

b) Offspring production

We analysed the cumulative number of offspring produced over the 17-day mating period by fertile flies in heat stressed and non-stressed treatments. This investigates if the heat-stressed flies that maintained fertility have lower lifetime reproduction than non-stressed flies. We removed permanently sterile individuals from this dataset, because we cannot be certain that counts of 0 are generated by the same biological process as variation in integer counts. Also, any variation in the ratio between 0 counts and non-0 counts is captured in our

analysis of fertility above, so including completely sterile individuals in analysis of offspring number partially re-reports this previous result (for offspring counts including 0s see Fig S4). Our sample sizes for offspring counts were: benign females=29, stressed females=42, benign males= 39, stressed males=22.

We tested the effect of heat treatment independently for males and females because focal males had four females to produce offspring with but focal females oviposited alone. As offspring number is typically female-driven, it is inappropriate to directly compare the two sexes – however this experimental design was necessary to maximise our detection of fertility. We used generalised linear models with quasi-Poisson distributions because of the count nature of the data and because Poisson model residuals were overdispersed.

c) Operational sex ratio

We combined our data on male and female complete sterility curves (see (a) above) over time to predict the OSRs for our temperature treatments. We did this by calculating the proportion of fertile males by the total proportion of fertile adults. This inherently corrects for any difference sin sample sizes in male and female treatments and allows for a potential 1:1 sex ratio. When calculated this way, the OSR can range from 0% where only females are fertile in the population, to 100% where only males are fertile in the population (Kvarnemo and Ahnesjo 1996). Because these predicted sex ratios are the product of our total observed data, we do not have variance with which to statistically test deviation from the expected 0.5. Rather, this serves as an illustration of the effect of heat on OSR.

Results

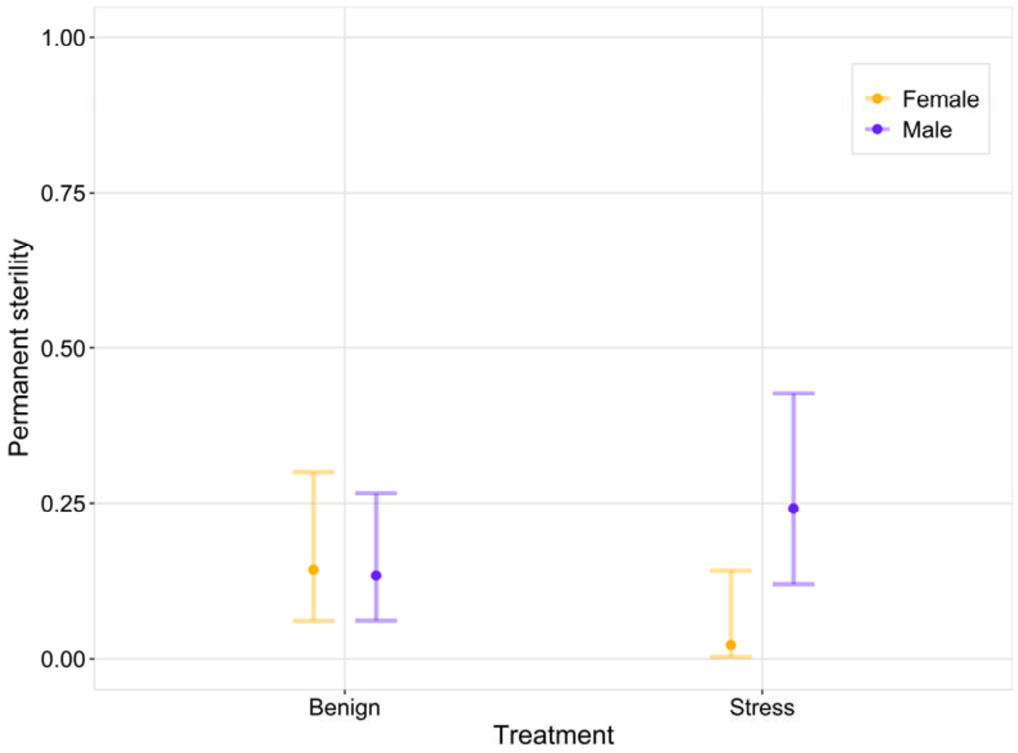

Figure S1. Proportion of male and female *D. virilis* that produced no offspring at all during the experiment (up to 17 days post-eclosion). Individuals were either kept at benign temperatures (23°C), or stressed (4h at 38°C) during the pupal stage. Error bars are 95% confidence intervals. Males exposed to heat stress were more likely than controls or females in any heat treatment to be rendered permanently sterile ($\chi^2_{(1)}$ = 5.657, p=0.017). Sample sizes: benign males= 45, stressed males=29, benign females=35, stressed females=45.

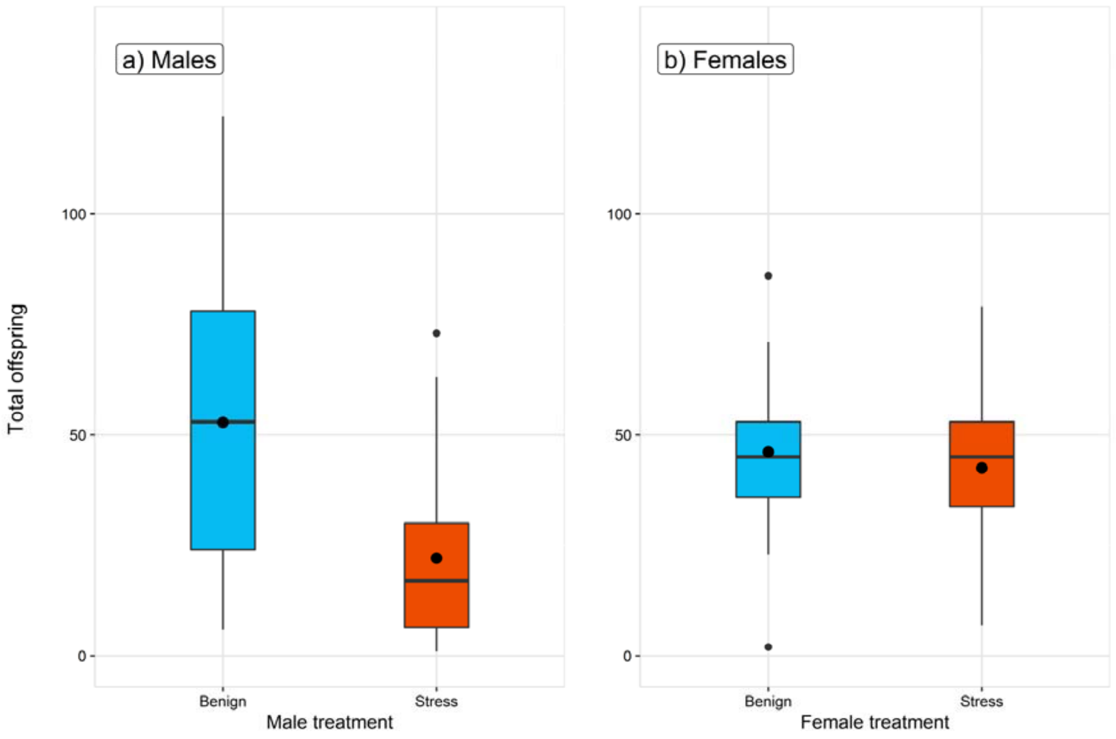

Figure S2. Total cumulative offspring number produced (laid or sired) by fertile focal individuals throughout the course of the experiment, when paired with 4 partners for 17 days. Black dots inside boxplots represent mean offspring number for each treatment. Individuals were either kept at benign temperatures (23°C) or stressed (4h at 38°C) during the pupal stage. In males, pupal heat stress significantly reduced pupal offspring number by 58% (estimate= -0.870, $t_{(59,1)} = -3.925$, $p < 0.001$). In females we find no significant effect of temperature stress on pupal offspring number (estimate= -0.081, $t_{(69,1)} = -0.928$, $p > 0.05$) Sample sizes: benign males= 39, stressed males=22, benign females=29, stressed females=42.

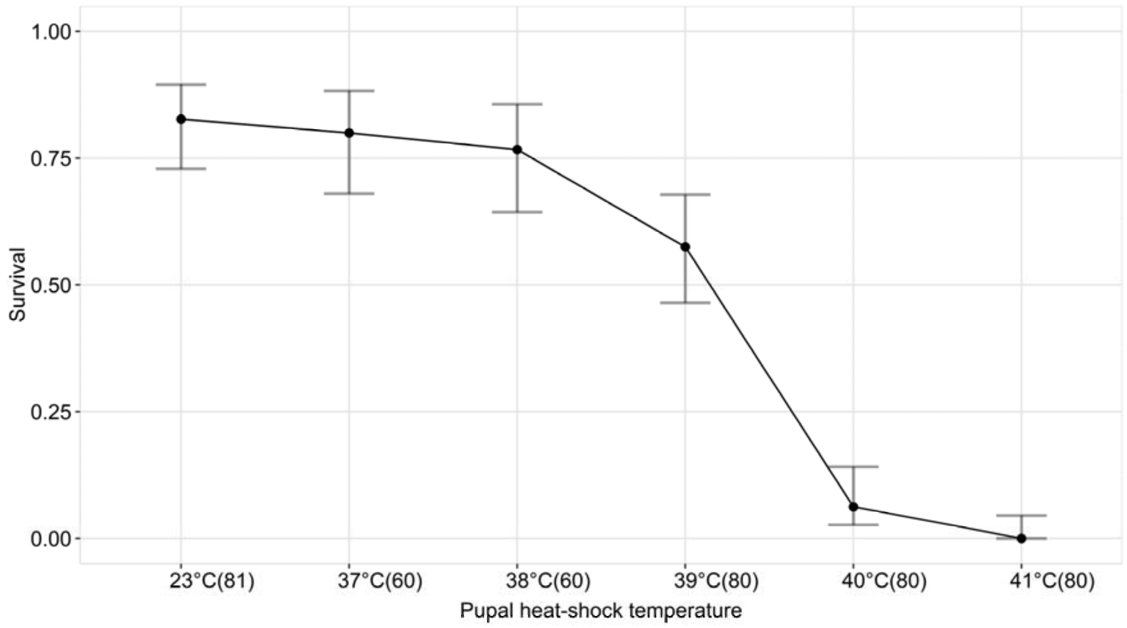

Figure S3: Preliminary data demonstrating the proportion of pupae that eclose after a 4h heat stress across a range of temperatures. We used these data to select 38°C as our stress temperature for the fertility assays because as it was the highest temperature that did not impose mean pupal survival below the 95% confidence intervals of survival at benign (23°C). Error bars are 95% confidence intervals. Pupal survival was assayed by heating pupae in the same way as described in the methods, but across 6 temperature treatments. Because it is difficult to record pupal death immediately following stress, we left flies to develop for 2 weeks and the number of eclosing adults were counted. Errors are 95% confidence intervals calculated with a logit link in the "binom" R package (Dorai-Raj 2014). Sample sizes are presented in brackets on the x-axis labels.

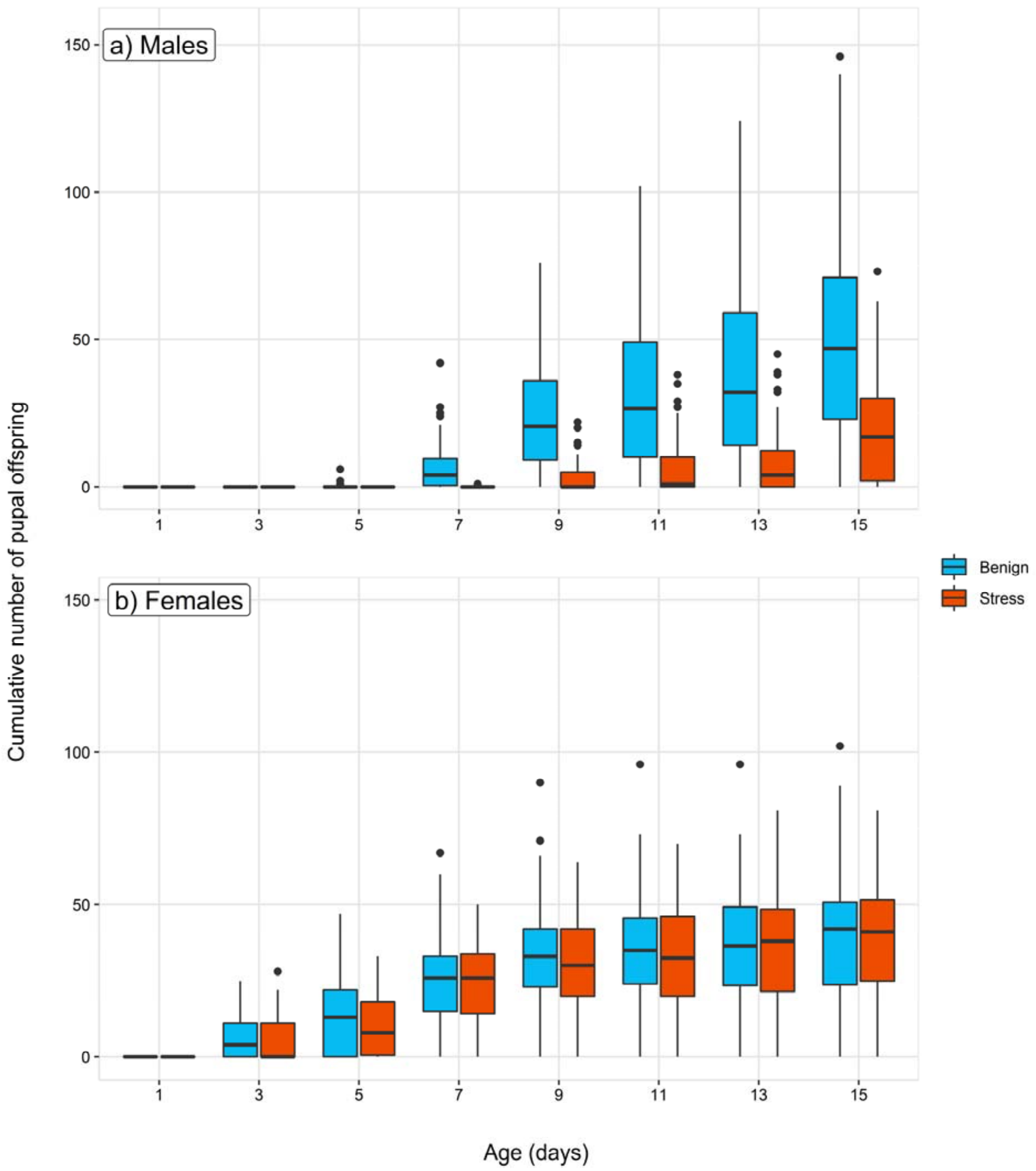

Figure S4: Cumulative offspring numbers produced (laid or sired) by a) male and b) female focal individuals at each measured time-point, when paired with 4 partners. Individuals were either kept at benign temperatures (23°C) or stressed (4h at 38°C) during the pupal stage. We include counts of 0 here to illustrate how recovery of fertility happened in males but they do not recover lifetime offspring production. Sample sizes: benign males= 45, stressed males=29, benign females=35, stressed females=45.

References

Dorai-Raj S. 2014. Binom: Binomial confidence intervals for several parameterizations. Version 1.1-1.

Fox JW, Sanford. 2011. An r companion to applied regression. Sage Thousand Oaks (CA).

Jørgensen KT, Sørensen JG, Bundgaard J. 2006 Heat tolerance and the effect of mild heat stress on reproductive characters in drosophila buzzatii males. Journal of Thermal Biology 31(3):280-286.

Kvarnemo C, Ahnesjo I. 1996 The dynamics of operational sex ratios and competition for mates. Trends in Ecology & Evolution 11(10):404-408.

Mirol PM, Routtu J, Hoikkala A, Butlin RK. 2008 Signals of demographic expansion in drosophila virilis. BMC Evolutionary Biology 8(1):59.

Pitnick S, Markow TA, Spicer GS. 1995 Delayed male maturity is a cost of producing large sperm in drosophila. Proceedings of the National Academy of Sciences 92(23):10614-10618.

Rohmer C, David JR, Moreteau B, Joly D. 2004 Heat induced male sterility in drosophila melanogaster: Adaptive genetic variations among geographic populations and role of the y chromosome. Journal of Experimental Biology 207(16):2735-2743.

Therneau T. 2015. A package for survival analysis in s. Version 2.42-3.

Wickham H. 2016. Ggplot2: Elegant graphics for data analysis. Springer-Verlag New York.
